# Predicting trail-making test performance using 7-Tesla structural cerebral MRI

**DOI:** 10.1038/s41398-026-04402-x

**Published:** 2026-10-19

**Authors:** Gereon J. Schnellbächer, Ravichandran Rajkumar, Tanja Veselinović, Shukti Ramkiran, Jana Hagen, Maria Collee, N. Jon Shah, Irene Neuner, Jürgen Dammers

**Affiliations:** 1 Department of Psychiatry, Psychotherapy and Psychosomatics, RWTH Aachen University, Aachen, Germany; 2 Institute of Neuroscience and Medicine 4, INM-4, Forschungszentrum Jülich, Jülich, Germany; 3 Department of Psychiatry and Psychotherapy II, LVR-Klinik Köln, Köln, Germany; 4 Department of Neurology, RWTH Aachen University, Aachen, Germany; 5 Department of Psychiatry, University of Southern Denmark, Odense University Hospital, Odense C, Denmark; 6 Present Address: Department of Psychiatry II, Ulm University and BKH Günzburg, Günzburg, Germany

**Keywords:** Predictive markers, Physiology

## Abstract

Psychopharmaceutical therapy has seen a lack of improvement in efficacy, highlighting the need for a more individualized treatment approach that optimally leverages pharmaceutical effects. To achieve this, it is necessary to identify factors that can predict psychological attributes and treatment outcomes. We hypothesize that brain morphology may serve as a predictor of performance in psychological tests. A 7-Tesla-MRI system was used to measure 74 transdiagnostic patients and 41 healthy controls. Values for grey matter volume (GMV), cortical thickness and gyrification based on the Schaefer 100 Atlas for parcellation were determined. Using this morphological data, performance in a Trail-Making Test (TMT) - A and B was predicted with the help of a deep learning model. A SHAP analysis was conducted to identify the key variables important for the model. The accuracy in predicting TMT performance was very good at 0.795. The most decisive feature influencing predictions was gyrification. Areas indicating inter-group differences were focused in the frontal cortex, as well as in temporal and parietal regions. Morphological characteristics can predict test performance with high accuracy. These results support the implementation of a transdiagnostic approach in clinical practice to better address the underlying pathophysiology. Symptom prediction based on neuroimaging data appears to be an achievable goal.

## Introduction

Psychopharmaceutic intervention is a cornerstone in the treatment of many psychiatric diseases and is often indispensable for symptom amelioration. However, its effectiveness has stagnated in recent decades [1]. This stagnation can be attributed to the enormous complexity and opacity of the cerebral processes that underlie both healthy physiology and pathological conditions. For the successful advancement of neuroscientific research, it is necessary to leverage the latest developments in the fields of both data acquisition and data analysis. Using an ultra-high field strength 7-Tesla (T)-MRI system, we aimed to ascertain the maximum amount of information contained within the structural features of the cerebrum while capitalizing on the advancements in MRI technology. Moreover, standardized psychological evaluations based on objective and reliable tests provide sophisticated and structured data that can be correlated with cerebral morphology. We anticipate that combining brain structural data with psychological assessments will lead to a better understanding of how disease manifests. Previous studies have delineated correlations between psychological test results and morphological or functional brain alterations at the group level [2, 3]. However, translating these insights into therapeutic improvements has been challenging, since they are not reliably reproducible at an individual level. To date, there is no known method to predict what class of antidepressants will effectively lead to an amelioration of symptoms in a specific case [4]. Identifying the most appropriate therapy for an individual patient would clearly be highly valuable. Advancements in data analysis, exemplified i.e. by modern deep learning models (DLMs), have the potential to provide personalized prognoses based on biomarkers [5]. A significant benefit of DLMs is their high scalability due to efficient algorithms that require relatively few computational power, albeit with the potential of low model transparency [6]. Data sets of moderate size are quite well interpretable with more intelligible models like a support vector machines (SVM). The future of medicine will, however, be most likely be defined by large data collections, where high computation costs would be a true constraint. This study used the morphological characteristics of a transdiagnostic group to predict the relative time necessary to complete a Trail-Making Test (TMT)-A and -B [7]. It employed a strict data-driven approach that does not rely on any a priori hypothesis regarding brain function. Every area of the Schaefer Atlas 100 [8] was included for each investigated volume and surface parameter – namely, grey matter volume (GMV), cortical thickness (CT) and gyrification. These features reflect both dynamic and static aspects of cerebral structure [9–12]. A supervised DLM using a triple loss function was employed [13, 14]. Despite the high number of observed features, no explicit dimension reduction steps were planned due to the model’s inherent ability to embed the relevant information in a smaller latent space [6]. This further reduces computational costs in contrast to i.e. SVMs that rely more on dimension reduction methods to avoid overfitting. The backpropagation and loss-function calculations typical of deep learning models are powerful tools for identifying complex patterns hidden within brain morphological data [15]. There is, however, a risk that the test-training split leads to a high variance in the accuracy of the outcome. Some brain scans may contain more usable information than others, potentially creating an overly optimistic view of their classification power. This was addressed through using an outer hold-out loop with ten random splits, for which we report the mean accuracy and standard deviation across all iterations. To assess model convergence and verify the absence of overfitting, training and validation loss curves were monitored across all ten hold-out iterations. Furthermore, Shapley Additive Explanations (SHAP) represents an advanced method for calculating the relative importance of individual variables within a model [16]. This approach was employed to maximize transparency, which is a prerequisite for clinical applicability. SHAP values were calculated based on the averages of all ten calculated models, ensuring the generalisability of our findings.

We hypothesise that artificial intelligence (AI) algorithms will be able to predict cognitive performance based on a combination of volumetric and surface parameters as measured by an ultra-high resolution 7T-MRI system. This study aimed to enhance the knowledge of the predictability of objective psychological parameters, independent of specific pathologies, based on biological markers at an individual level. It also tries to introduce deep learning methods into clinical research through addressing the problem of generalizability with the 10 outer hold-loops and the problem of transparency with the SHAP-analysis [17, 18]. Such research is of critical importance for developing more individualized therapy concepts, maximizing the effectiveness of existing therapies and potentially leading to significant advances in patient care over the coming decades.

## Methods

### Recruitment

Seventy-four patients undergoing treatment at the Universitätsklinik Aachen were recruited for the study, with a mean age of 32.08 (standard deviation = 11.44). The patient group included 30 cisgender women and one transgender man (assigned female at birth and receiving gender-affirming hormone therapy). The healthy control group comprised 41 individuals with a mean age of 29.00 (standard deviation = 10.52), including 15 cisgender women. There were no significant between-group differences in gender distribution (chi-square, *p* = 0.676) or age (t-test *p* = 0.158). Thirty-three patients and 25 controls demonstrated a TMT-A completion time faster than the sample median of 25.169 s. Two patients were excluded from TMT-B analysis due to insufficient knowledge of the Latin alphabet. Twenty-eight patients and 28 controls had a TMT-B time faster than the median of 55.928 s. Out of the 74 patients, 49 had major depressive disorder, 8 were diagnosed with schizophrenia, 5 had alcohol abuse disorders, 2 had attention deficit hyperactivity disorder (ADHD), 3 had psychosis of a non-schizophrenic origin, 3 were diagnosed with drug abuse and 4 had bipolar disorder. Healthy participants had no current or lifetime neurologic or psychiatric disorders, as assessed with the Mini International Neuropsychiatric Interview (M.I.N.I.) [19]. The data sample with 115 measurements in total was considered adequate based on the previous experience of the authors with MRI-based prediction models [5].

Handedness was determined using the Edinburgh Handedness Inventory [20], and only right-handed participants were included in the study. All volunteers received financial compensation for their participation and additional travel expenses, if applicable. Participants with MRI contraindications or unstable medical conditions on the day of the scan were excluded. All psychological measurements were conducted within a week of the MRI scan. Informed written consent was obtained from all participants prior to the investigation. The study protocol was approved by the local Ethics Committee of the Universitätsklinik Aachen. All methods were performed in accordance with the relevant guidelines and regulations and in line with the Declaration of Helsinki.

### MRI parameters

MRI data acquisition was performed with a 7T Magnetom Terra scanner (Siemens Healthineers, Erlangen, Germany) equipped with a 1Tx 32Rx Head Coil 7T Clinical from Nova Medical (Wilmongton, MA, USA) at the Institute of Neuroscience and Medicine (INM-4), Forschungszentrum Jülich. The MP2RAGE sequence was employed. MP2RAGE is a variation of the standard magnetization-prepared rapid gradient echo (MPRAGE) sequence. It acquires two gradient echo images with different inversion time (TI) and flip angle (FA) (inversion image 1 (INV1) TI = 840 ms, flip, FA = 5°, INV2 IT = 2370 ms, FA = 6°). The other sequence-related parameters were similar for both gradient echo images: echo time (TE) = 1.99 ms; repetition time (TR) = 4500 ms for signal-to-noise ratio (SNR) optimization. The image matrix was set to 320 × 300 with a 0.75 mm isotropic resolution in 208 sagittal slices. The T_1_-weighted anatomical images referred to here were produced by combining the two gradient echo images by means of a ratio, as explained by Marques et al. [21]. The combined image was free from proton density contrast, T2* contrast, reception bias field and first-order transmit field inhomogeneity.

### Preprocessing

The raw Digital Imaging and Communications in Medicine (DICOM) scans were converted to 3D T1-weighted Neuroimaging Informatics Technology Initiative (NIfTI) format, using MRIcron software (https://www.nitrc.org/projects/mricron). The 3D T1-weighted images were visually audited to check for scan quality, artefacts and abnormal tissues using FSLeyes View software (https://fsl.fmrib.ox.ac.uk/fsl/fslwiki/FslView). VBM was performed using v7771 software (FIL Methods Group, University College London, London, UK) implemented in MATLAB R2023a v9.14 (The MathWorks, Natick, MA, USA). VBM preprocessing was performed using the CAT12.9-toolbox in expert mode, with inhomogeneity correction set to strong.

Following brain extraction, a registration process was applied to ensure that the voxel positions matched the anatomical positions. In order to align the image to the Montreal Neurological Institute (MNI) space, a spatial transformation was performed through affine registration. Affine registration has 12 degrees of freedom in 3-dimensional space, namely three rotations, three translations, three scalings and three skew/shears. This linear registration is necessary in order to remove, for instance, movement artefacts. It also initializes the non-linear registration, which is indispensable for between-subject group comparisons. In the transformation, a 4 × 4 matrix is multiplied by the coordinate vectors in order to define the “warped” coordinates in the deformation field. The CAT toolbox uses the geodesic approach [22] for registration. A geodesic represents the shortest path between two points on a curved surface. In this context, the algorithm searches for the shortest path between the coordinates of two brain images in a complex high-dimensional space. This approach expands on affine registration by allowing more nuanced, non-linear transformations. Differences between the images are expressed through a cost function, which the algorithm aims to minimize. Problems with radio frequency (RF) coil uniformity, eddy currents or the anatomy of the measured subject may cause artefact-like bias [23]. After registration, the CAT toolbox uses the unified segmentation approach, in which higher computing costs are exchanged for better accuracy [24]. Here, bias correction, registration through non-linear warping, and segmentation are done in a single step. This is supported by tissue probability maps in which an a priori likelihood for the presence of a particular tissue at a given voxel is delineated from existing neuroimaging data. For region definition, the Schaefer Atlas was used [8], whereby areas were defined according to functional connectivity patterns. The entire brain was partitioned using a k-means algorithm with k = 100. These 100 areas demonstrate similar connectivity, and their extent is thus strictly data-driven.

Following the voxel-based processing, the surface-based processing steps were performed. Employing a projection-based thickness (PBT) method [25], the cortical thickness and gyrification estimation and reconstruction of the central surface were completed in single step. Subsequently, the topological defects were corrected using spherical harmonics [26] followed by surface refinement, resulting in the final central surface mesh. The individual central surfaces are spatially registered to the ‘FsAverage’ template of Freesurfer, using a spherical mapping with minimal distortions [27]. Finally, the local thickness and gyrification values were transferred onto the ‘FsAverage’ template.

The segmented images were inspected visually for non-brain structures by overlaying GM segments on the structural image for each subject. Additionally, the CAT toolbox provided automated image and preprocessing quality scores for each subject. Image quality before preprocessing was assessed via a weighted overall image quality score accounting for resolution, noise and bias.

### Psychological testing

The TMT consists of two parts: TMT-A and TMT-B [7]. TMT-A includes 25 numbers (1-25) arranged in circles spread out on a sheet of paper. The task is to connect all numbers with a line without lifting the pen from the paper. TMT-B is similar, but features 13 numbers (1-13) and 12 letters (A-L) that also have to be connected in sequence. The added challenge of TMT-B is that the logical order of 1-A, 2-B, etc. has to be followed. TMT performance has been shown to correlate with working memory, psychomotor speed, and executive function [28]. Although errors in the test are not explicitly measured, they are considered in the interpretation, as the tester often corrects the participant, thereby increasing the time needed to complete the task [29]. The total amount of time taken, measured in seconds, serves as the interpretable raw data of the assessment.

### Classification

For training and classification, we used the same model structure as described in [14]. The model is a feed-forward neural network that employs a triple loss objective function, coupled with a softmax activation function. The number of input features used was 304, with the first and second hidden layers containing 64 and 32 units, respectively.

Training data were input into a directed and acyclic network, which processes the data through various layers to classify the measurements into the two categories: above-median and below-median TMT-completion time. Since we employed a supervised learning algorithm, this prediction was tested during model optimization using a labelled data matrix.

**Fig. 1:**
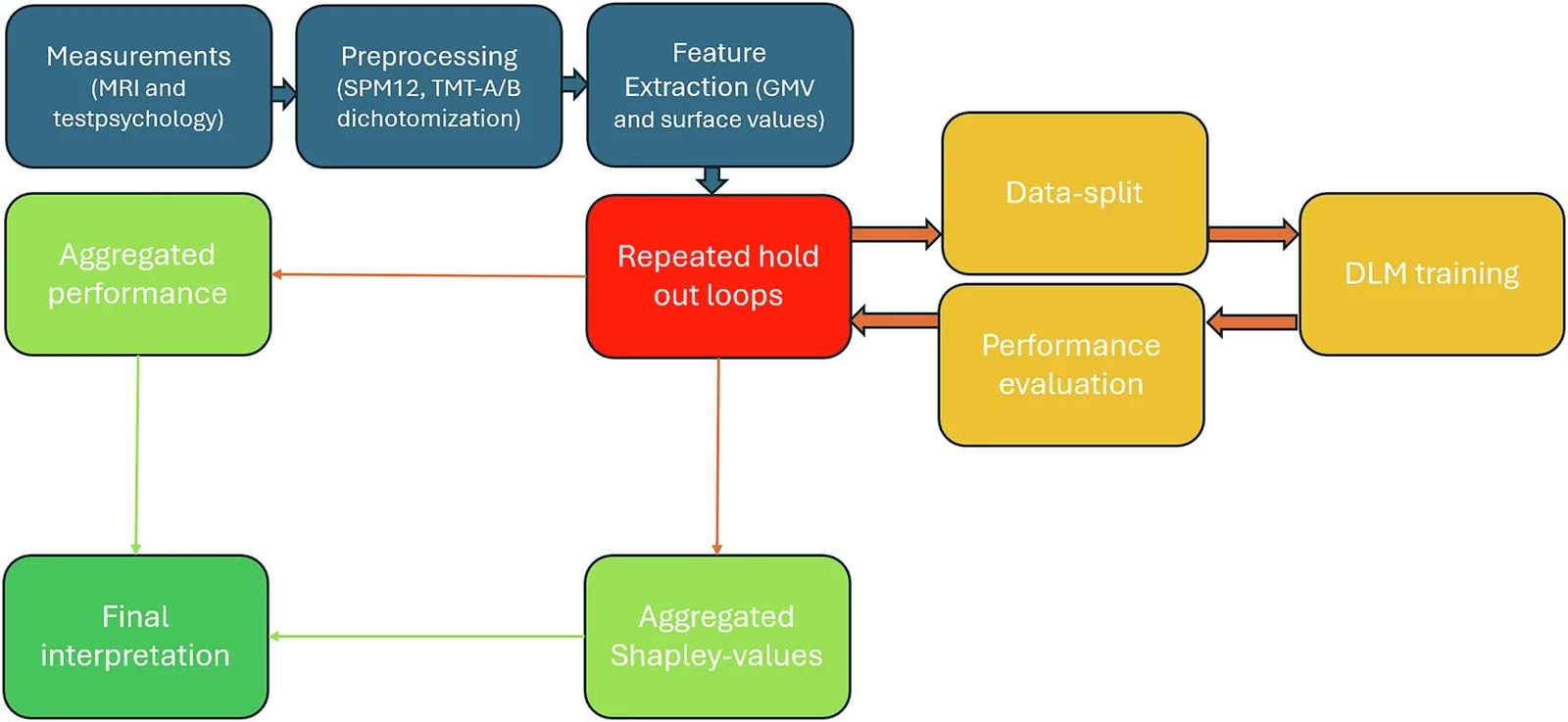
Schematic overview of the analysis pipeline. Structural variables are evaluated through the repeated hold‑out loops comprising data splitting, deep learning model (DLM) training and performance evaluation. Across all iterations, model performance metrics and Shapley values are aggregated.

Some brain scans may contain more usable information than others, potentially creating an overly optimistic view of their classification power. To address this, ten iterations of a hold‑out loop were located upstream of the model in the data flow and executed using ten random splits of the training data (Fig. 1). To minimize the loss, the gradients of the model parameters were computed through backpropagation. The model was trained for a maximum number of 200 epochs. The optimizer, Adaptive Moment Estimation (Adam), updated the parameters based on these gradients with a learning rate of 0.2 [30], while a learning-rate scheduler (ReduceLROnPlateau. (factor 0.5, patience 5, minimum learning rate 1 × 10-6)) [31], reduced the learning rate if the validation loss stopped improving. Gradient clipping was applied with a maximum norm of 1.0. This iterative process continued until the model converged. Early stopping was not employed. The network used a two‑layer fully connected architecture with batch normalization and a triple loss function. The triple loss function was selected for its ability to do predictions even in limited data sets with a relatively low risk of overfitting. Crucially, to ensure a rigorous evaluation and prevent data leakage, the data splitting process utilized a strict Leave-Subject-Out (LSO) approach. No subject’s data was simultaneously present in both the training and validation sets during any given hold-out iteration, ensuring that the model’s performance reflects true generalization to unseen patients. Overfitting was further controlled through the repeated holdout validation, learning‑rate scheduling, gradient clipping, and a fixed training budget across all runs.

In a subsequent step, a SHAP summary plot was delineated using the SHAP package in Python to visualize the contribution of each variable to model performance [16]. The delineated values reflect the variance contained in the data points transmitted to the DLM. These values were calculated as an average across all ten hold-out split iterations to ensure maximum generalizability. Data and code will be made available on request.

## Results

**Fig. 2:**
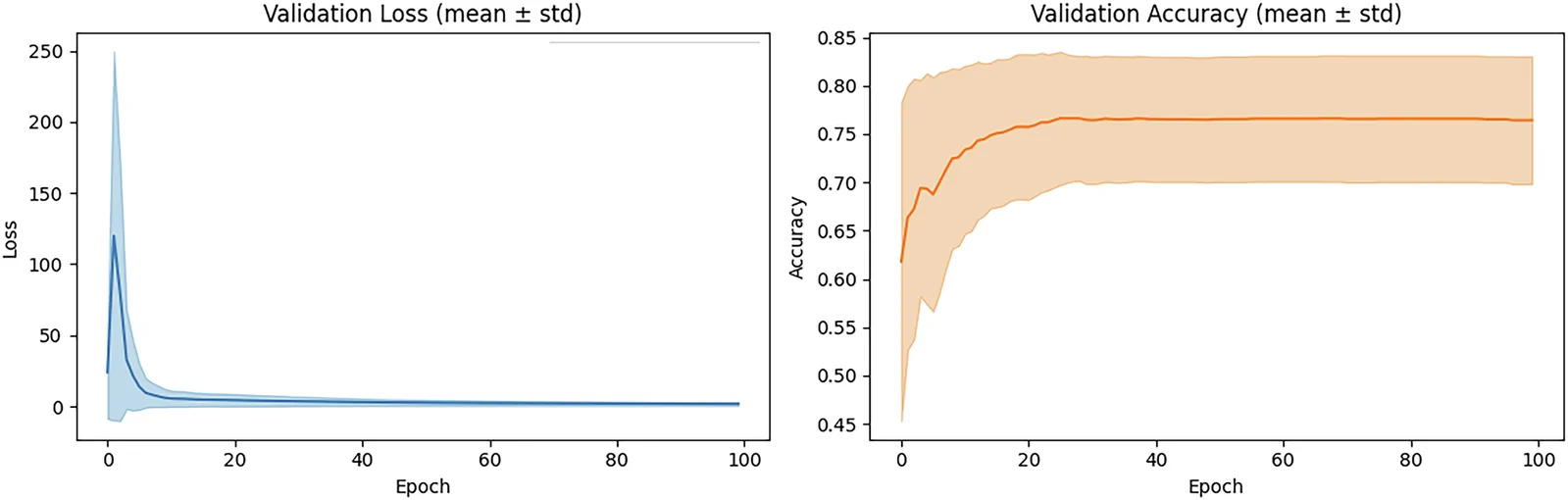
Validation loss and accuracy functions across repeated hold‑out runs. Curves showing the mean validation loss and mean validation accuracy per epoch across all iterations, with shaded areas indicating the standard deviation.

After calculating ten iterations of a DLM with varying train-test-split randomization seeds, we were able to delineate a very good average accuracy of 0.795 and a median accuracy of 0.795. The lowest recorded accuracy was 0.682, while the highest reached 0.891. The model achieved in the final iteration a validation loss of 0.602 and a validation accuracy of 0.773, suggesting an imperfect but good classification performance. As illustrated in Fig. 2 the validation loss tracked the training loss closely throughout the epochs, with accuracy reaching a stable plateau by approximately epoch 30–40. The relatively low standard deviation across iterations (accuracy standard deviation = 0.068) further corroborates stable model behaviour. These observations indicate that the model learned generalizable patterns without fitting to noise in the training data.

**Fig. 3:**
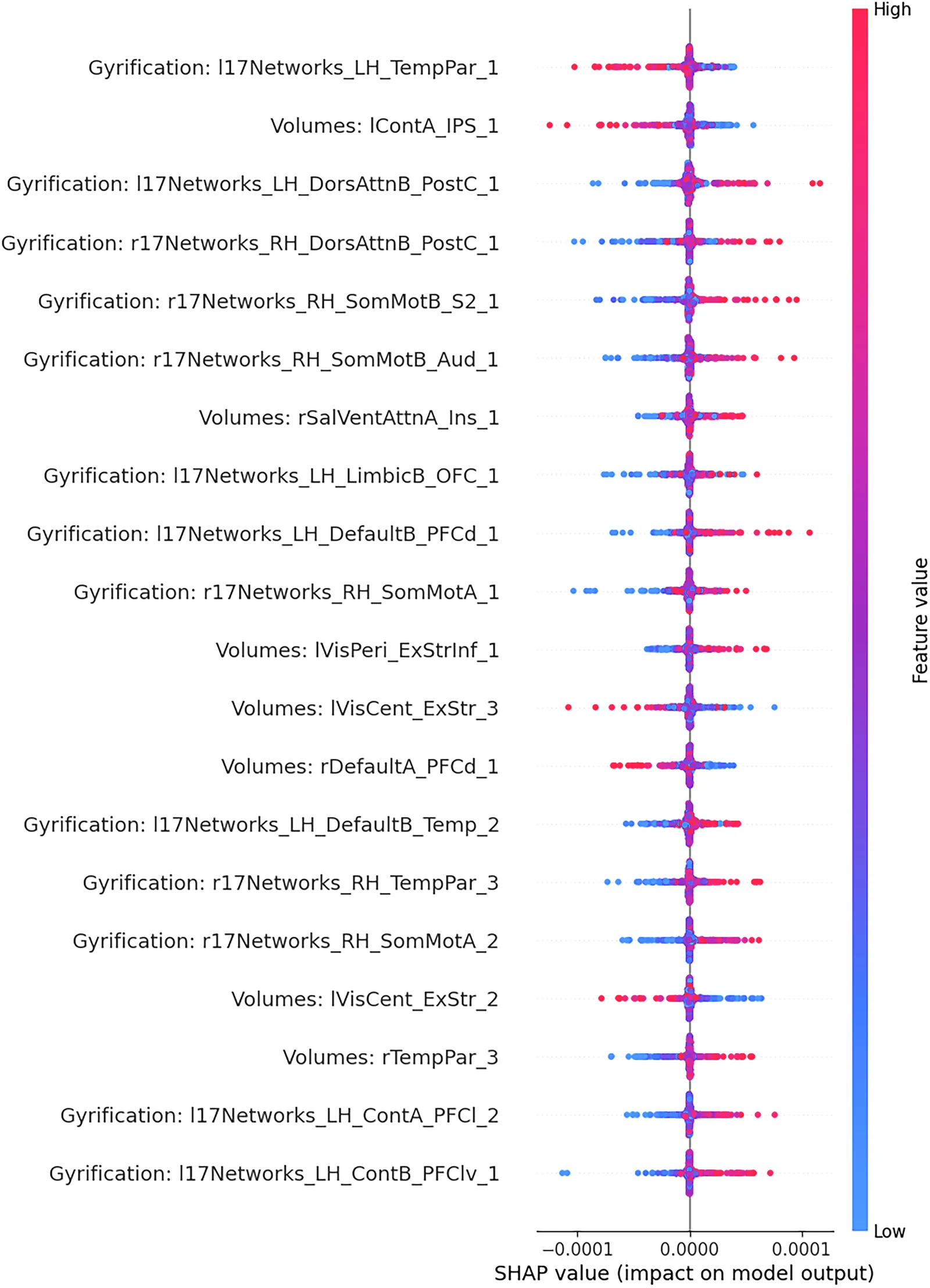
Waterfall plot illustrating SHAP values for the most influential features in the predictive model. Each bar represents an individual feature’s contribution to the model output, with red indicating higher feature values and blue indicating lower feature values. Positive SHAP values push the prediction toward a higher outcome estimate, whereas negative SHAP values decrease it. Key neuroanatomical features shown include gyrification and grey matter volume.

**Fig. 4:**
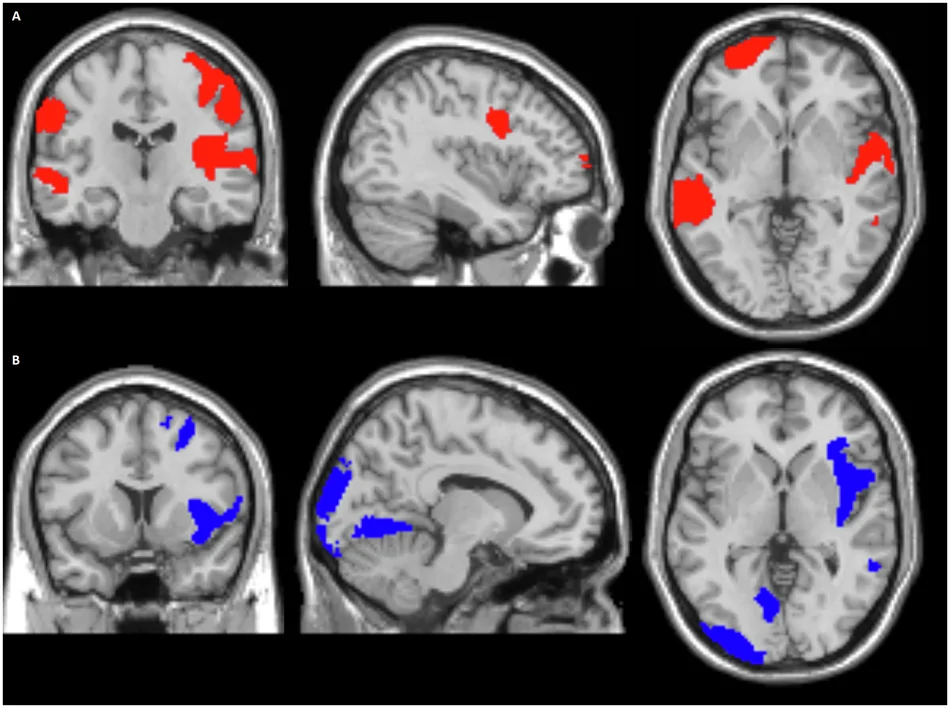
Brain images illustrating regions most influential for model performance as identified by the SHAP analysis. **A** Gyrification features contributing to the model are shown in red. **B** Grey matter volume features are displayed in blue.

The features that influenced model performance most strongly were gyrification and the GMV of several different regions like the parietal, frontal and occipital cortex. The gyrification measurements from the sensorimotor network (SMN), limbic network, the default mode network (DMN) and the dorsal attention network (DAN) provided a relatively high degree of predictive information. Specific regions of the SMN were the auditory cortex, while the limbic network delineated alterations in the OFC. The DMN was represented with the dorsal PFC and temporal cortex, while the DAN demonstrated gyrification alterations in the postcentral cortex (Figs. 3, 4).

## Discussion

The application of a DLM produced reliable classification outcomes, distinguishing participants with above- and below-median TMT completion times based solely on the morphological configuration of the cerebrum. Through SHAP analysis, it was possible to see which regions affected the classification results, leading to greater transparency and enabling a detailed interpretation. These results support the notion that advancements in data acquisition and analysis may establish a foundation for more personalized therapy approaches in psychiatry.

### Cerebral morphology as a predictor of cognition

In our study, we combined the TMT-A and TMT-B measurements to enhance the intra-subject variability during model training and improve model accuracy. Importantly, while the number of features used remained unchanged, the sample size per subject was doubled, leading to increased model reliability due to a more optimal data point/feature number ratio. Variance was quite evenly distributed across variables, with no single brain area showing dominant predictive power, as indicated by the low spread of Shapley values. The relatively low standard deviations across all measurements after the repeated hold-out loops further support the generalizability of our results.

Through SHAP analysis, we identified areas in the SMN, limbic network, DAN and DMN. The inclusion of the somatomotor system seems intuitive, as test completion requires a high level of motor coordination and the ability to fine-tune muscle strength and speed [7]. In a computerized version of the TMTs, there was an increased activation in the right inferior/middle frontal cortices and the precentral gyrus [32]. Moreover, a verbal adaptation of the TMTs, which omitted the confounding activation of motor areas, delineated an activation asymmetry in the left dorsolateral PFC and supplementary motor area/cingulate sulcus [33]. This indicates a significant role of the dorsolateral PFC and the ACC, independent of motor function. Fittingly, a lesion mapping study showed that GMV damage in areas such as the dorsolateral PFC and anterior cingulate cortex negatively impacts the ability to perform TMTs [34]. The gyrification and the GMV of the dorsal PFC as well as the gyrification of the lateral PFC contributed to classification accuracy reflecting these previous results .

The limbic system played an important role in model performance with the gyrification of the left OFC. The OFC is well-known for its role in temporal cognition and sequencing, which are essential for successful test completion [35]. It is also associated with the ability to process the temporal sequence of stimulus and reward [36]. Its direct link with TMT-performance is emphasized by changes in functional connectivity in comparison to a fixation task in the gyrus rectus within the OFC [37]. In our analysis, the DAN was primarily represented through the postcentral gyrus. Similarly, in a study examining the white matter integrity of the DAN in unsafe drivers, a correlation between axial diffusivity values in the superior longitudinal fasciculus and worse TMT-A scores was found [38]. A group comparison between patients with type II diabetes and healthy controls showed significantly worse TMT-A measurements for the diabetes group. This coincided with decreased functional connectivity in the right precuneus of the DMN and in the left inferior parietal lobule within the DAN, as well as higher inter-network connectivity between the DAN and DMN. These findings may serve as a potential biomarker for cognitive decline in diabetes patients [39]. Typically, DMN activation is anticorrelated with the DAN as they fulfil reciprocal roles in directing attention either inward or outward [40]. Psychomotor speed and working memory, as measured by the TMT-A, is associated with reduced sensorimotor afferents towards the DMN [41]. In older adults, slower TMT-B performance positively correlated with lower anterior DMN integrity [42]. In general, the TMT-B is more complex and cognitively demanding than the TMT-A [43]. One study used the TMT-B – TMT-A difference score as a measure of executive response switching and demonstrated lower performance for participants with structural damage within the left rostral anterior cingulate cortex [44]. Similarly, a study based on a regression analysis highlighted cognitive flexibility as the primary distinction between TMT-B and TMT-A performance [45]. Structural alterations in the left dorsomedial prefrontal lobe, including CT differences in controls or lesions in neurological patients, are associated with poor TMT-B performance [46]. The role of the DMN in TMT tasks is well documented [47]. The dorsal PFC is known for its involvement in the TMTs [48], which is – as stated above – the very DMN-region also delineated by the SHAP-analysis as important for the model. The abundance of previous literature confirming abnormalities in these specific areas and networks, as identified by the DLM, validates the reliability our data-driven approach.

GMV, CT and gyrification differ in their dynamics and should be interpreted separately in order to gain an adequate understanding of the underlying processes. In adults, GMV is subject to changes over time to a much higher degree than gyrification [9, 10, 49, 50], as is CT [11, 12]. This susceptibility to change may make GMV and CT more useful for delineating temporary changes compared to gyrification. For instance, a long-term reduction in cognitive capacity due to chronic stress stretching back for years would not strongly influence the degree of cortical folding [51]. The DLM indicated a high reliance on gyrification in comparison to GMV. The feature of CT did not appear in the list of most important areas at all (Fig. 3). This suggests that predisposing structural patterns are primarily determined by ontogenesis and early childhood development. However, the GMV of the temporal or parietal-occipital cortex were also relevant for the model’s performance, indicating that modifiable structural features still have an impact.

### Classification in psychiatry – by symptoms or by diagnosis?

A sizable proportion of both the healthy subjects and the patients in the study demonstrated above median TMT-A and TMT-B times, indicating that diagnosis was not a reliable predictor of low cognitive function as measured by the TMTs. This supports the case for a transdiagnostic approach based on tangible measurements. Structural features, psychological test results, demographic background information and other data should inform clinical decisions. Symptoms experienced by patients with a specific diagnosis can vary significantly. It is reasonable to assume that such varying clusters of symptoms are caused by different underlying processes. This is supported by a study that proposed four different “biotypes” of depression based on functional connectivity profiles [52]. A transdiagnostic approach is much more flexible and able to address the actual symptomatology of the individual patient as they present at that moment, without needing to categorize them into broad categories, thus avoiding a loss of information. Additionally, this approach may offer advantages for future research in psychopharmacology. Currently, study populations are selected based on diagnosis, which results in problems associated with the inherent heterogeneity of these diagnoses [53]. Important features, such as the aforementioned “biotypes”, are often overlooked, potentially diminishing treatment effectiveness. For the effective research of biological therapy methods, it is necessary to identify somatic features associated with psychopathology to bridge the gap between clinical phenomenology and apparent bodily health, which is commonly seen in psychiatric diseases.

The model’s prediction accuracy was among others influenced by regions such as the OFC of the limbic system, which is usually associated less with executive function and motor control, but affective disease [54, 55]. The TMTs were chosen explicitly for their ability to report on executive function, which has a high physiological variability in a healthy population, but which is also affected by various different pathologies. Thus, the TMTs were assumed to be able to reflect on a broad range of individual variance. The fact that our heterogeneous sample of measurements could be classified with such high accuracy seems to confirm this hypothesis. The delineated pattern of complex morphological alterations, overlapping with areas changed by pathology, highlights the fluid nature of the brain as a functional system, where problems in one area can also lead to decreased performance in other areas. This systems-level perspective may also explain why different psychiatric conditions—such as bipolar disorder, unipolar depression, and the negative symptoms of schizophrenia—can present with indistinguishable phenotypic profiles. However, systematically capturing these complex interactions requires a high degree of information and exceeds the capacity of traditional analytical approaches. Therefore, data analytical tools like AI are essential to understand the underlying processes involved. Further, dimensional descriptions of pathology are necessary to better reflect on the complexity of the underlying processes. This study successfully used a DLM to predict psychological performance with high accuracy based on brain structure. Establishing such an association is a crucial first step in finding variables able to predict which biological interventions are most likely to improve cognitive function and could perspectively help address the challenge of limited advancements in the effectiveness of psychiatric therapies over recent decades.

## Conclusion

DLMs can accurately predict below-median and above-median TMT-performance based solely on the morphological configuration of the cerebrum. The data structure showed a relatively even distribution of variance, with no single brain area serving as the determining factor. While gyrification was by far the most significant feature predicting TMT measurements, the models also relied on GMV for classification. The regions showing inter-group differences included the temporo-parietal and frontal cortices, consistent with prior research. Our results support a transdiagnostic, non-categorical classification approach that aims to better reflect on intricate pathophysiologies and demonstrate the feasibility of DLM employment in a clinical setting.
